# A *KRT6A* mutation p.Ile462Asn in a Chinese family with pachyonychia congenita, and identification of maternal mosaicism: a case report

**DOI:** 10.1186/s12920-021-01109-4

**Published:** 2021-11-01

**Authors:** Yue Li, Yumeng Wang, Yan Ming, Pan Chaolan, Zhang Jia, Ni Cheng, Cao Qiaoyu, Ming Li, Xu Tianyi

**Affiliations:** 1 Departments of Dermatology, Xinhua Hospital, Shanghai Jiaotong University School of Medicine, 1665 Kongjiang Road, Shanghai, 200092 China; 2 Center for Rare Disease, Shanghai, China; 3 Department of Obstetrics, The International Peace Maternity and Child Health Hospital, School of Medicine, Shanghai Jiao Tong University, 910 Henshan Road, Shanghai, 200030 China; 4 Department of Dermatology, Huashan Hospital, Fudan University, Shanghai, 200040 China

**Keywords:** Pachyonychia congentia (PC), Keratin 6A, Mosaicism, Whole exome sequencing, SNaPshot sequencing, HiSeq deep sequencing

## Abstract

**Background:**

Pachyonychia congenita (PC, OMIM #167200, #167210, #615726, #615728, and #615735) is a rare autosomal dominant disorder caused by keratin gene mutations in *KRT6A,KRT6B,KRT6C,KRT16* or *KRT17*. It is characterized with nail dystrophy and palmoplantar keratoderma (PPK). The most prominent manifestation is plantar pain. This is a further unusual case of parental mosaicism in PC. Although very rare, germ cell mosaicism should be considered when providing genetic counselling for unaffected parents of a child with PC.

**Case presentation:**

We report the case of a 5-year-old boy with thickening nails and oral leukokeratosis at birth. He began to develop palmoplantar keratoderma at 2 years old and his sister has similar clinical manifestation characterized with nail discoloration and thickening. A previously reported heterozygous mutation, p.Ile462Asn, was identified in KRT6A in the proband and his affected sister. SNaPshot sequencing revealed mosaicism at a level of 2.5% and 4.7% in DNA from blood and hair bulbs from the unaffected mother. HiSeq deep sequencing demonstrated low-grade mosaicism in the patient’s younger sister and parents.

**Conclusion:**

These findings indicate the ability of WES and SNaPshot sequencing to detect low-frequency mosaic mutations. Although very rare, germinal mosaicism should be considered when genetic counseling is given to families with presumed spontaneous cases of PC.

## Background

Pachyonychia congenita (PC, OMIM #167200, #167210, #615726, #615728, and #615735) is a rare autosomal dominant disorder caused by keratin gene mutations in *KRT6A, KRT6B, KRT6C, KRT16 or KRT17* [1]. It is characterized with nail dystrophy and palmoplantar keratoderma (PPK). The most prominent manifestation is plantar pain [2]. Additional characteristics can include oral leukokeratosis, epidermal inclusion cysts, pilosebaceous cysts, follicular keratoses, hyperhidrosis and sometimes natal teeth [3].

In this study, we identified a KRT6A mutation, p.Ile462Asn, in a Chinese PC family with two affected children with unaffected parents. Using whole exome sequencing (WES) and SNaPshot sequencing, we confirmed inheritance by maternal mosaicism. To our knowledge, this is a further unusual case of parental mosaicism in PC, this time occurring in a woman.

## Case presentation

**Fig.1:**
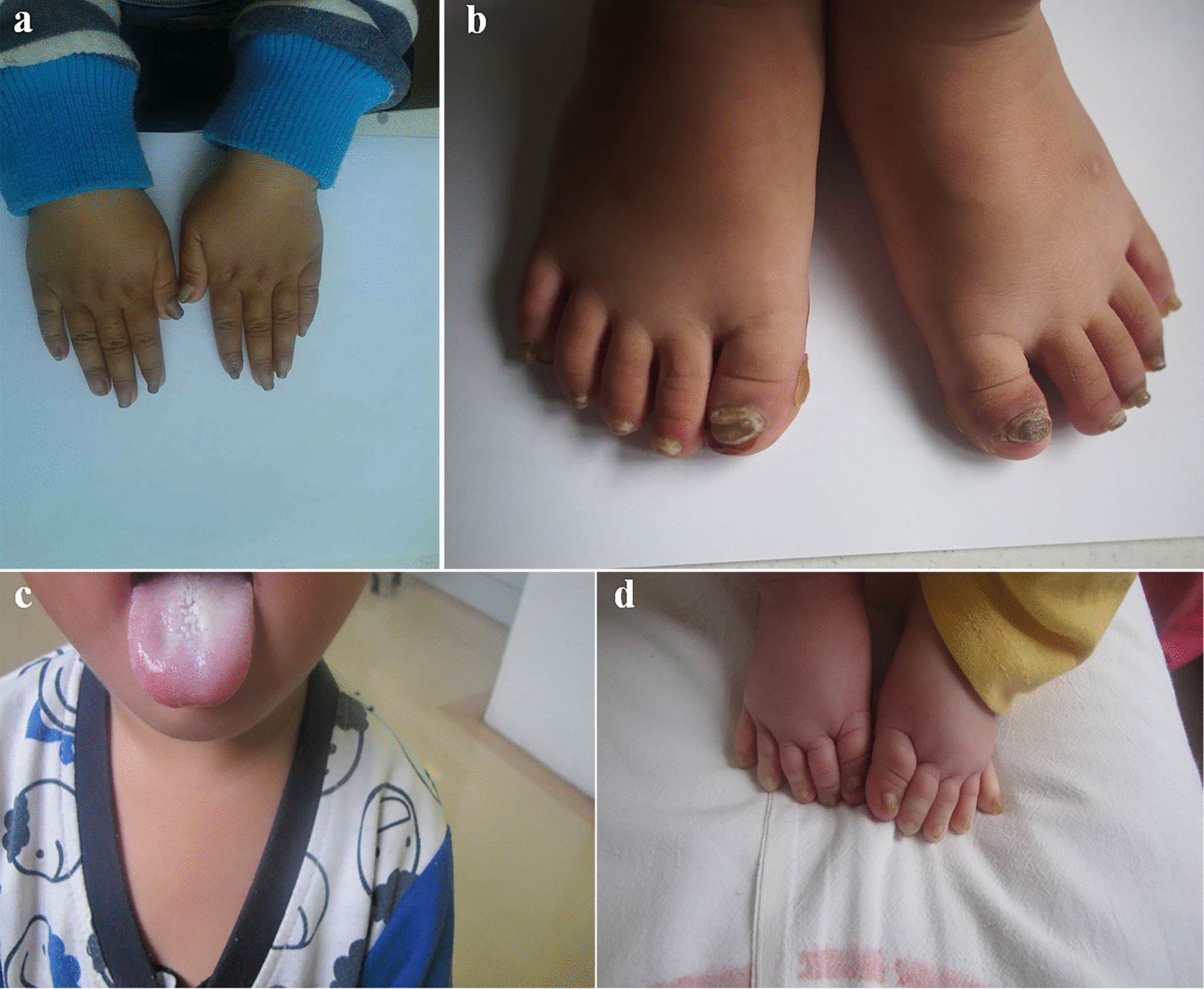
**a**, **b** Hypertrophic nails of the proband. **c** Oral leucokeratosis of the proband. **d** Hypertrophic nails of the proband’s sister

The proband of this family is a 5-year-old boy from Zhejiang province in China. He developed thickening nails and oral leukokeratosis at birth (Fig. 1a–c), and began developing palmoplantar keratoderma at 2 years old. His sister had similar clinical manifestation characterized with thickening nail and discoloration (Fig. 1d). No abnormalities in the teeth and eyes were noted in the two affected children. There were no unaffected siblings, And the phenotypic features of PC were not found in any other family members including their parents.

After informed consent, genomic DNA was extracted from the peripheral blood lymphocytes of this family. DNA was also extracted from hair bulbs, buccal smears and sperm cells of the proband’s father and hair bulbs and buccal smears of his mother using a QIAGEN QIAamp Blood Mini kit. This study was approved by the Ethics Committees of Shanghai Jiaotong University School of Medicine and conducted in accordance with the principles of the Declaration of Helsinki.

*KRT6A, KRT6B, KRT6C, KRT16* and *KRT17* genes of this family were analyzed by direct sequencing using primers and reaction conditions as previously described. In addition, samples from 100 unrelated population-matched controls were sequenced to exclude the possibility that the variant was a polymorphism in the *KRT6A* gene (GenBank accession number: NM_005554.3).

The exome capture were performed using Agilent SureSelect Human All Exon Kits (Agilent, Santa Clara, CA) according to the manufacturer’s instructions. Sequencing was performed on a HiSeq 2000 platform with read lengths of 100 bp. The mean coverage depth for each sample is 100 × . The sequencing reads were described according to NCBI human reference sequence.

**Fig. 2:**
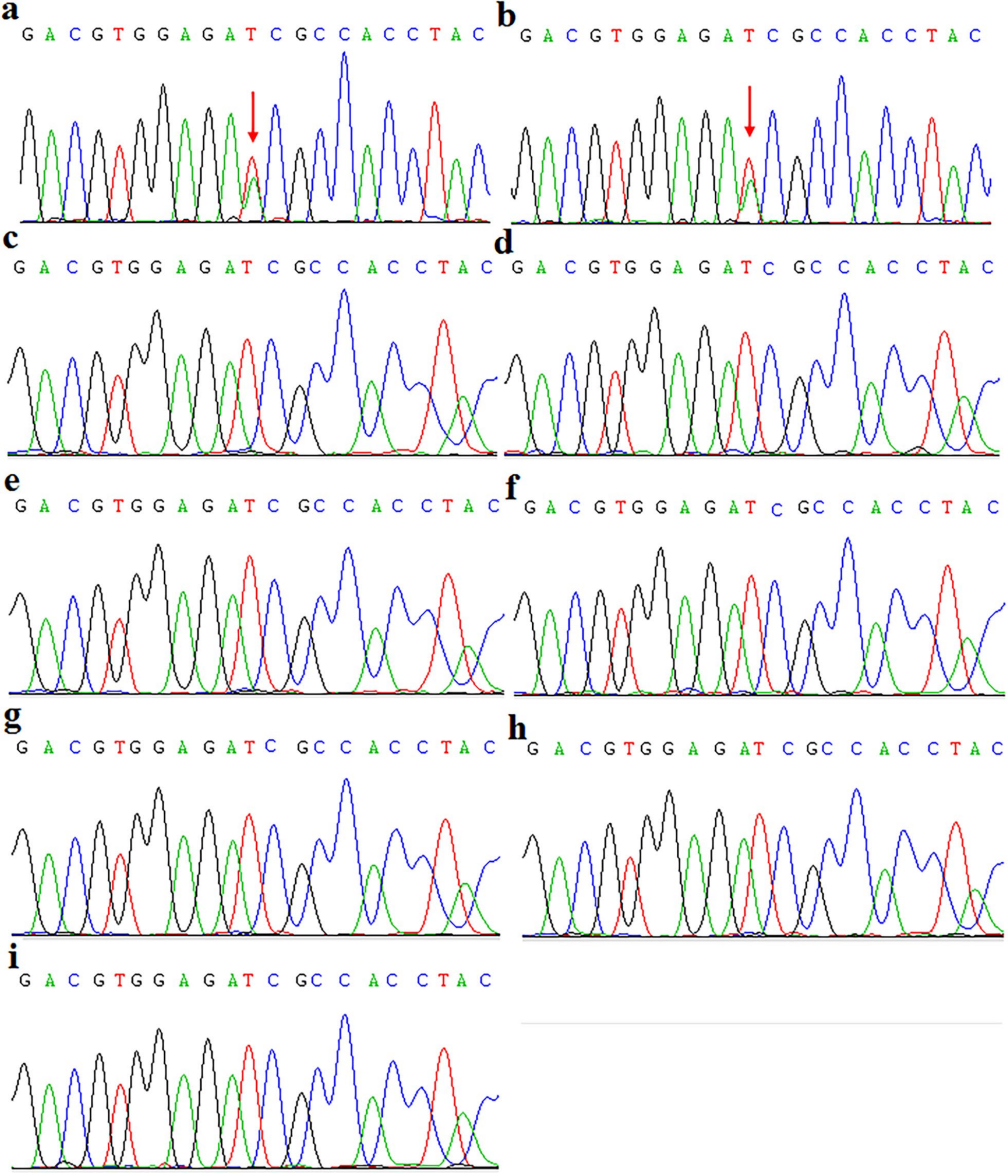
**a** Sequencing analysis revealed a heterozygous c.1385T > A transition in exon 7 of *KRT6A* in the proband’s genomic DNA from blood. **b** Sequencing analysis revealed a heterozygous c.1385T > A transition in exon 7 of *KRT6A* in his sister’s DNA from blood. **c**, **d** The sequence of DNA derived from the parents’ blood, **e**–**f** hair bulbs and **g**, **h** buccal smears was wild-type. **i** No mutation was identified in father’s sperm cells

**Fig. 3:**
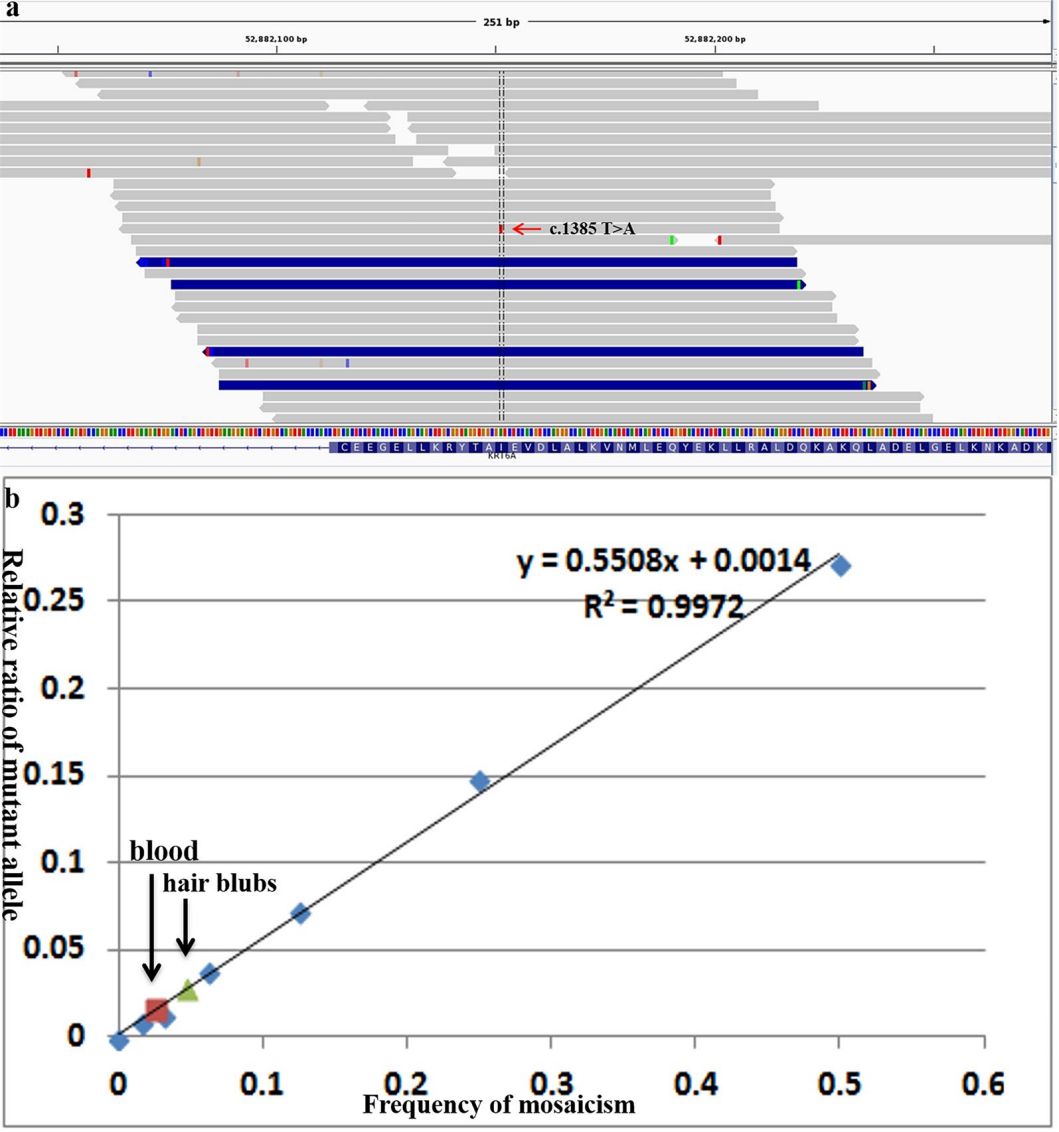
**a** A heterozygous c.1385T > A transition in exon 7 of *KRT6A* was identified by WES from mother's blood. The red arrow indicates the variant c.1385T > A. **b** Standard curve of the mutant allele quantity, derived from serial dilutions of DNA from a heterozygous patient and a normal control, in which 50%, 25%, 12.5%, 6.25%, 3.13%, and 1.56% of the DNA has a mutation. SNaPshot sequencing revealed mosaicism at level of 2.5% and 4.7% in the mother’s DNA from blood and hair bulbs

The entire coding and flanking intronic sequences of *KRT6A, KRT6B, KRT6C, KRT16* and *KRT17* genes were screened for mutations in the two affected children and unaffected parents. A previously reported heterozygous mutation, p.Ile462Asn, was identified in *KRT6A* in the proband and his sister (Fig. 2a, b). This change was not detected in 100 unrelated, healthy Chinese control individuals (200 alleles). Sequence analysis of the four other keratin genes failed to detect sequence variants in either affected or unaffected individuals of the family (Fig. 2c, d). This mutation was not identified in the parents, in DNA derived from peripheral blood, hair bulbs or buccal smears (Fig. 2e–h). The sperm cells from proband’s father were also wildtype (Fig. 2i). Since the two affected children harbored the same pathogenic mutation, we postulated that one of the parents was mosaic for this variant WES was performed on the two affected children and their parents. Approximately 5 billion bases were sequenced with coverage of 100 × . Consequently, the variant was detected in one sequencing read from 86 sequencing reads from DNA derived from the mother’s blood (Fig. 3a). The mutation was not identified in DNA derived from the father’s blood by whole exome sequencing. The frequency of reads was 47% and 49% in proband and his sister, respectively. The results indicated that the mutation in KRT6A may be from maternal mosaicism in this family.

To confirm the question of somatic mosaicism in the mother, the analysis was performed to quantify the proportion of cells carrying the KRT6A mutation by using SNaPshot (ABI Prism SNaPShot multiplex kit; Applied Biosystems) on an ABI PRISM 3730 genetic analyser according to the manufacturer’s instructions. The proportion of normal and mutant DNA was quantified using GeneMapper software (v4.0; Applied Biosystems). To get mutation ratios of 50%, 25%, 12.5%, 6.25%, 3.13%, and 1.56%, a genomic DNA sample of a heterozygous proband was serially diluted with a sample of a wild-type family member. All experiments were repeated three times.

SNaPshot analysis revealed substantial mutation-level variation in the two affected children and their parents. SNaPshot sequencing revealed mosaicism at level of 2.5% and 4.7% in DNA from the mother’s blood and hair bulbs (Fig. 3b). No mosaicism was identified in DNA from buccal smears from mother. A non-mosaic wild-type state was found in the healthy father (Data not shown).

Besides, we also performed HiSeq deep sequencing. Firstly, We diluted the DNA from the patient’s blood with the DNA from the normal by 1/2, 1/4, 1/8, 1/16, 1/32, 1/64, 1/128 based on gradient dilution method. Making a standard curve. Then, We designed primer detection mutation site KRT6A (NM_005554.3) c.1385T > A; p.Ile462Asn. (F:TTCCTCTTCCAGTGCGCCAA; R:AGCTGTTGAAGGAGKT CGTGT) And synthesizing fusion primer. (F1:ACACGACGCTCTTCCGATCTT TCCTCTTCCAGTGCGCCAA; R1:TTCCTTGGCACCCGAGAATTCCAAGCTG TTGAAGGAGKTCGTGT) Next step, We carried out the first round PCR. (3min96°C; 15 cycles of 30 s 96 °C; 30 s 60 °C; 30 s 72 °C. End with 5 min incubation at 72 °C; pause at 10 °C.) And the second round were carried out after screened and purified. (NNNNNN was used to distinguish between different samples. F2:AATGATACGGCGACCACCGAGATCTACACTCTTTCCCTACA CGACGCTCTTCCGATCT; R2-x:CAAGCAGAAGACGGCATACGAGATNNN NNNGTGACTGGAGTTCCTTGGCAC CCGAGAAT) (3min96°C; 10 cycles of 15 s 96 °C; 30 s 60 °C; 30 s 72 °C. End with 5 min incubation at 72 °C; pause at 10 °C.) In the end, Sequencing the PCR products from last step after purified by Illumina Hiseq. And analyzing the number of T and A at the site to be tested in the total read length of each sample.

We sequencing the DNA sample from patient’s younger sister and parents in the same method. Calculating by Y = 1.0007x − 0.0036 as Table 1.

**Table 1:** The number of T and A at the site to be tested in the total read length of patient’s family members

Sample	Ref	Alt	Ref	Alt	Total	Alt detection ratio (X) (%)	Alt calculating ratio (Y) (%)
DNA from patient’s younger sister	T	A	99,254	98,266	199,990	49.14	48.8099
DNA from blood of patient’s father	T	A	197,737	226	199,986	0.11	− 0.2469
DNA from blood of patient’s mother	T	A	192,109	5917	199,992	2.96	2.6007
DNA from mouth mucosa of patient’s father	T	A	141,449	162	143,068	0.11	− 0.2467
DNA from mouth mucosa of patient’s mother	T	A	197,106	961	199,988	0.48	0.1209
DNA from hair blubs of patient’s father	T	A	13,098	47	13,265	0.35	− 0.0054
DNA from hair blubs of patient’s mother	T	A	181,637	11,293	195,019	5.79	5.4348
DNA from sperm of patient’s father	T	A	171,327	187	173,214	0.11	− 0.2520

HiSeq deep sequencing demonstrated low-grade mosaicism in the patient’s younger sister and parents

## Discussion

Although very rare, germline mosaicism had been confirmed by molecular diagnosis for some dominant diseases, including keratin disorders epidermolysis bullosa simplex (EBS) and PC as well as dystrophic epidermolysis bullosa pruriginosa, and Ehlers-Danlos syndrome type IV [4–6]. In 2011, Pho et al. reported the first case of germ cell mosaicism in PC [7]. There were two affected children with unaffected parents in this family. The authors confirmed the pathogenic mutation p.Asn172del in *KRT6A* gene from the unaffected father’s sperm cells. To date (April 2021), there were more than 1038 PC patients in the International Pachyonychia Congenita Research Registry (IPCRR, www.pachyonychia.org), that have genetically confirmed PC, this is the only family with germ cell mosaicism. Gu et al. reported a Japanese EBS patient with a de novo 1649delG mutation in *KRT5* gene in 2003 [8]. The parents were unaffected and the mutation was not detected in DNA derived from blood samples therefore it was reported as a de novo mutation [9]. However, the proband’s younger sister was revealed to be affected with EBS at birth in 2004. Further investigations demonstrated somatic and germline mosaicism in the mother of two affected children [5].

To our knowledge, this is a further unusual case of parental mosaicism in PC. When we observed two affected children with unaffected parents, one possible explanation was that it was an autosomal recessive disorder. Although there are known recessive cases for other keratin disorders such as epidermolysis bullosa simplex there are no recessive cases of PC with confirmed genetic analysis reported to date. One case previously reported as recessive PC has now been identified as PLACK syndrome with a mutation in the *CAST* gene [7, 10]. Other rarer possibilities to consider in these situations are germ cell mosaicism or paternal identity for accurate genetic counselling. Somatic mosaicism of a mutation in diseases with autosomal-dominant traits gives a few clinical manifestations but is not transmitted to future offspring. Whereas germ cell mosaicism of a mutation in autosomal dominant disorders does not present with a clinical phenotype but the disorder is transmitted to future offspring as observed in the reported cases of PC and neurofibromatosis 1. In some cases, individuals can have a mosaic mutation that affects germline and somatic cells as reported by Shen et al. [11]. A mother of a dystrophic epidermolysis bullosa patient who presented with a very mild DEB-blistering phenotype was confirmed to be a germline and somatic mosaic. In her skin, 28% of the pro-ɑ1 (VII) procollagen chains contained the mutation, which is higher than the threshold (10–25%) to develop disease, so her clinical manifestation was very mild, with a mild blistering phenotype [12]. Recently, Li et al. also reported a case of mosaic ichthyosis with different allele frequency in different tissues [13]. In our family, we demonstrate low-grade mosaicism in the mother, with a mutational load of 2.5% and 4.7% in her blood and hair bulbs, respectively. She is not affected. Her children harbouring a mutational load of 50% do express a phenotype. Apparently low-grade mosaicism is tolerated by the body, The threshold for developing PC-K6a must therefore be higher than 4.7% mutant *KRT6A*.

To determine the mosaic mutations, we use ultra deep sequencing, SNaPshot sequencing or pyrosequencing [14]. SNaPshot sequencing was the simplest method for confirmation of the frequency of mosaicism in this case. But it is difficult to detect mosaicism at a level of 2% or less [15]. Ultra deep sequencing and pyrosequencing are sensitive, but they are expensive. It is difficult to implement them in the routine diagnostic laboratory [16]. In our case, we confirmed maternal mosaicism down to 3% using 100 × whole exome sequencing and SNaPshot sequencing. These findings indicate the ability of whole exome sequencing, coupled with SNaPshot sequencing confirmatory analyses, to detect low-level mosaicism.

In summary, we report a recurrent p.Ile462Asn mutation in *KRT6A* gene in two children with PC with unaffected parents. This is a further unusual case of parental mosaicism in PC. Although very rare, germ cell mosaicism should be considered when providing genetic counselling for unaffected parents of a child with PC. We demonstrated that WES and SNaPshot sequencing can be useful technologies for confirmation of somatic and germinal mosaicism.

## Data Availability

The datasets and code generated during and/or analyzed during the current study are available in https://github.com/wuhanchun/BMC-materials.
